# Flexible and reconfigurable radio frequency electronics realized by high-throughput screen printing of vanadium dioxide switches

**DOI:** 10.1038/s41378-020-00194-2

**Published:** 2020-10-05

**Authors:** Weiwei Li, Mohammad Vaseem, Shuai Yang, Atif Shamim

**Affiliations:** IMPACT Lab, Computer, Electrical and Mathematical Sciences and Engineering (CEMSE) Divisiorn, King Abdullah University of Science and Technology (KAUST), Thuwal, 23955-6900 Kingdom of Saudi Arabia

**Keywords:** Electrical and electronic engineering, Nanoparticles

## Abstract

Smart materials that can change their properties based on an applied stimulus are in high demand due to their suitability for reconfigurable electronics, such as tunable filters or antennas. In particular, materials that undergo a metal–insulator transition (MIT), for example, vanadium dioxide (VO_2_) (M), are highly attractive due to their tunable electrical and optical properties at a low transition temperature of 68 °C. Although deposition of this material on a limited scale has been demonstrated through vacuum-based fabrication methods, its scalable application for large-area and high-volume processes is still challenging. Screen printing can be a viable option because of its high-throughput fabrication process on flexible substrates. In this work, we synthesize high-purity VO_2_ (M) microparticles and develop a screen-printable VO_2_ ink, enabling the large-area and high-resolution printing of VO_2_ switches on various substrates. The electrical properties of screen-printed VO_2_ switches at the microscale are thoroughly investigated under both thermal and electrical stimuli, and the switches exhibit a low ON resistance of 1.8 ohms and an ON/OFF ratio of more than 300. The electrical performance of the printed switches does not degrade even after multiple bending cycles and for bending radii as small as 1 mm. As a proof of concept, a fully printed and mechanically flexible band-pass filter is demonstrated that utilizes these printed switches as reconfigurable elements. Based on the ON and OFF conditions of the VO_2_ switches, the filter can reconfigure its operating frequency from 3.95 to 3.77 GHz without any degradation in performance during bending.

## Devices: Printing vanadium dioxide switches

Vanadium dioxide switches are screen printed in a high-throughput process. Vanadium dioxide is an attractive material for electrical devices as it displays a metal–insulator transition that can be controlled by thermal, optical, and electrical stimuli. However, producing vanadium dioxide devices over large areas and in high quantities is challenging. Here, a team from King Abdullah University of Science and Technology led by Prof. Atif Shamim reports a microparticles-based vanadium dioxide ink suitable for screen-printing. They print switches onto rigid and flexible substrates, achieving a high ON/OFF ratio of 300 and a low ON resistance of 1.8 ohm. After integrating the switches into band-stop filters and band-pass filters, the operation frequency of the filters can be reconfigured. This work demonstrates the possibility of printing vanadium dioxide for large-scale electronic devices.

## Introduction

In recent years, smart electronics have gained tremendous attention in both scientific and industrial fields^1^ due to their ability to transform, change their shape or tuning, and modulate their properties in response to external stimuli, such as mechanical deformation^2^, thermal heating^3^, an electrical field^4^, or a magnetic force^5^. Creating smart electronics and exploring their versatile applications in smart energy devices^4,6^, smart skins^7,8^, smart wearables^9–11^, reconfigurable electronics^12–14^, and even smart cities^15^, has raised new requirements for functional materials that can tune their properties according to specific demands^6,13,16,17^. Despite a diverse cluster of new materials, such as carbon-based materials^2,10^, transition metal dichalcogenides^18^, and metal oxide nanocrystals^19^, metal insulator transition (MIT) materials have great promise due to their easy and reversible tunability between the metal and insulator via various external stimuli^20–23^. For example, vanadium dioxide (VO_2_) (M) exhibits MIT behavior by possessing an insulating state at room temperature and a metallic state at a critical temperature (~68 °C)^24^ and is attracting a substantial amount of interest for optical and electrical electronic applications^12,17,25–29^. Currently, a variety of techniques, such as radio frequency (RF) sputtering^30,31^, pulsed laser deposition^32^, and electron beam evaporation^29^, have been evaluated to fabricate high-quality VO_2_ films with an ON/OFF ratio of over 10^4^. Unfortunately, these previously reported deposition methods operate under high vacuum conditions. They either consist of complex steps or deposit VO_2_ thin films with a limited size, which is not favorable for efficient fabrication. In addition, a high processing temperature is typically required during deposition, limiting compatibility with flexible polymer substrates.

Recently, printed electronics have made rapid progress. By depositing functional materials via conventional printing techniques (e.g., inkjet printing and screen printing), printed electronics offer a new method for achieving high-throughput and roll-to-roll production of electronic devices and integrated systems at low cost^33–38^. Despite the great progress made in printable materials and printing technologies, few reports have succeeded in developing printable VO_2_ ink to deposit high-quality VO_2_ films. Ji et al. prepared an inkjet-printable VO_2_ ink, fabricated VO_2_ films, and studied the infrared thermochromic properties^39^. Our group reported a VO_2_ nanoparticle-based ink for the inkjet printing of VO_2_ films. The printed VO_2_ films demonstrated an electrical conductivity of ~1 S m^−1^ in the insulating state and 150–200 S m^−1^ in the metallic state, resulting in a conductivity ratio of 10^2^^17^. The ink has also been used to fabricate RF switches, exhibiting decent RF performance from low frequencies (10 MHz) up to 40 GHz^12^, which is comparable to that of nonprinted VO_2_ switches^29^. However, these inkjet printable VO_2_ inks suffer from low VO_2_ loading, possible nozzle clogging, unstable printing, and slow deposition. The printed VO_2_ films exhibit poor adhesion to substrates and less flexibility due to the absence of polymer additives. In addition, to achieve improved electrical performance, thick films (tens of micrometers) are typically required; thus, tens or hundreds of printing passes are necessary to generate high-quality VO_2_ films, which is not favorable for high-efficiency fabrication, especially for large-area printing.

In this work, we synthesize high-crystal VO_2_ (M) microparticles and report a simple route to produce VO_2_ ink suitable for screen-printing techniques. With this ink, we demonstrate high-throughput printing of VO_2_ switches on both flexible polymers (Kapton and poly[ethylene terephthalate] (PET)) and rigid sapphire wafers with high resolution (down to 60 µm) at high speed (220 mm s^−1^) over a large area. The printed VO_2_ switches exhibit excellent electrical performance (ON/OFF ratio of 300 and conductivity as high as 1037 S m^−1^ after heating at 120 °C), excellent mechanical stability (bending radius down to 1 mm), satisfactory air stability (negligible performance change after 1 month), and decent RF performance (−1 dB isolation and −2.6 dB insertion loss up to 20 GHz). Finally, we demonstrate fully screen-printed reconfigurable RF electronic devices, including series switches, band-stop filters, and band-pass filters.

## Results

**Fig. 1:**
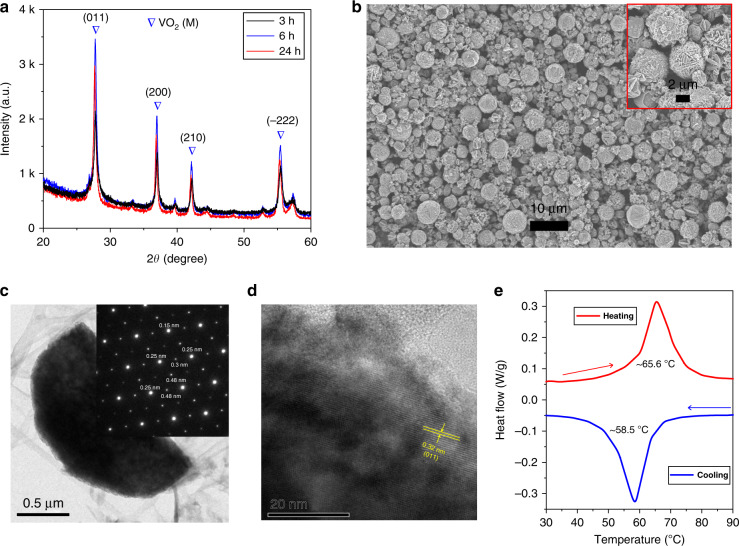
Characterization of the VO_2_ microparticles after annealing at 300 °C for 3 h in vacuum. **a** XRD spectra for the different reaction times, **b** SEM images, **c** TEM image and the corresponding SAED pattern, **d** HR-TEM image, and **e** DSC spectrum

In our previous study^17^, we determined that as-prepared VO_2_ (M) nanoparticles do not have high quality due to a low crystallinity, which resulted in poor electrical performance. In this work, we prepared highly crystalline VO_2_ microparticles using an autoclave reactor in an aqueous environment using a commercially available V_2_O_5_ powder (≥98%, Sigma-Aldrich) as the precursor and oxalic acid as the reducing agent. Initially, X-ray diffraction (XRD) analysis and morphological observations were conducted for V_2_O_5_ powder, as presented in Fig. S1. It is clearly shown that the V_2_O_5_ powder consisted of micron-sized particles, and multiple crystalline peaks were observed in the XRD pattern. We designed experiments using a constant weight percentage of precursor materials with different reaction times (i.e., 3, 6, and 24 h), and the products were investigated by XRD analysis. As displayed in Fig. S2, the as-synthesized samples were a mixture of undesired VO_2_ (A) and desired VO_2_ (M) phases that produced low-intensity XRD peaks. To achieve a pure VO_2_ (M) phase, we exposed the as-synthesized samples to different annealing environments. The XRD spectra demonstrated in Fig. 1a clearly reveal that a pure VO_2_ (M) phase was obtained for all the samples synthesized for 3-, 6- and 24‑h reaction times after annealing at 300 °C in vacuum. The XRD peaks observed at 27.84°, 37.01°, 42.19°, and 55.56° were indexed to the [011], [200], [210] and [−222] crystal planes, which is consistent with JCPDS No. 72–0514. It is interesting to note that the sample synthesized for 3 h followed by annealing demonstrated low-intensity XRD peaks, while the 6- and 24‑h reaction time samples demonstrated higher intensity peaks. Thus, VO_2_ particles synthesized with a reaction time of 6 h and an annealing temperature of 300 °C were used for further study. It is noteworthy that the reaction time is much shorter than those reported in the literature, where more than one day has been used for the synthesis^40–42^.

The as-annealed products were further characterized for their morphological and thermal behavior. It can be seen in the scanning electron microscopy (SEM) image in Fig. 1b that the VO_2_ particles were uniformly grown and comprised micron-scale sheets that were arranged in a flower-shaped morphology, with an average particle size from 4–6 µm (inset in Fig. 1b). It is interesting to note that the morphology of the as-annealed products is similar to that of the V_2_O_5_ precursor powder. Thus, the shape and size of the VO_2_ particles can be further optimized by selecting the finest V_2_O_5_ powder with regular sizes either from commercially available sources or through custom in-house preparation of V_2_O_5_ particles. A micron-thick sheet was also characterized by transmission electron microscopy (TEM), as illustrated in Fig. 1c, d. The inset in Fig. 1c displays a selected-area electron diffraction (SAED) pattern of the sheet, which confirmed its crystalline nature with interplanar distances of 0.15, 0.25, 0.3, and 0.48 nm. The high-resolution TEM (HR-TEM) image displayed in Fig. 1d indicates that the distance between two crystal planes was 0.32 nm, which corresponds to (011) crystal planes. The existence of an MIT temperature is an attractive property of VO_2_, and it was assessed via thermal analysis using differential scanning calorimetry (DSC), as displayed in Fig. 1e. The annealed VO_2_ particles were exposed to heating and cooling from room temperature to 90 °C. Two clear MIT peaks at ~65.6 °C during heating and ~58.5 °C during cooling are observed. The calculated thermal width was ~7 °C, which corresponds to a first-order phase transition. The thermal width is lower than that of previously reported VO_2_ nanoparticles (~18 °C)^17^ because of the high quality of the as-annealed VO_2_ particles, which is of importance in smart optical and electrical switches^43^. It should be noted that the MIT temperature is an inherent property of the VO_2_ (M) phase, and its bulk value is ~68 °C. In our case, we observed that the MIT temperature of the as-annealed VO_2_ particles is slightly lower than that of the bulk VO2 particles, which is most likely due to the impurity of the precursor (i.e., V_2_O_5_, ≥ 98%) used for the synthesis. It is well known that impurities and doping have prominent effects on the MIT temperature, and even 1–2% impurities/doping can decrease the MIT temperature^44–46^. We observed that the MIT temperature of the as-synthesized VO_2_ particles (without annealing) was not distinct because the phase was not in a pure state, as confirmed by the DSC spectrum in Fig. S3a. Once the pure VO_2_ (M) phase was achieved after annealing, similar MIT temperatures were observed. This was confirmed by the sample with a long reaction time of 24 h, where an MIT temperature of 65.69 °C was obtained (Fig. S3b).

**Fig. 2:**
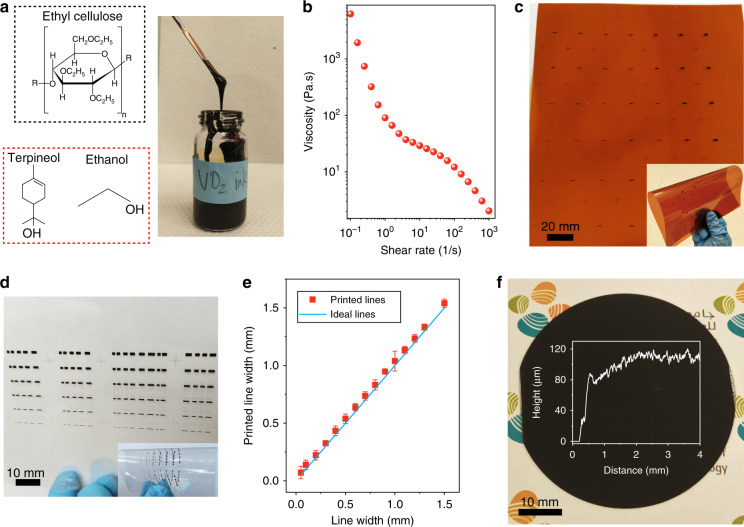
VO_2_ ink properties and large-area printing of VO_2_ switches. **a** Molecular structures of binder (ethyl cellulose) and solvents (terpineol and ethanol) and digital photograph of the prepared VO_2_ ink. **b** The measured viscosity of the VO_2_ ink at a shear rate from 0.1 to 1000 s^−1^. **c** Digital photographs of the screen-printed VO_2_ switches on a Kapton substrate. Inset: Bent VO_2_ switches. **d** Digital photographs of the screen-printed VO_2_ switches on a PET substrate. Inset: Bent VO_2_ switches. **e** The printed VO_2_ line width as a function of the designed line width on the screen mesh. **f** Digital photograph of the screen-printed VO_2_ film on a 2-inch sapphire wafer. Inset: profilometry data from the printed VO_2_ layer

It is clear from the results in Fig. 1 that pure VO_2_ (M) particles were obtained. To develop a screen-printable VO_2_ ink, a mixed solvent of terpineol and ethanol (Fig. 2a) was selected due to its high viscosity and low surface tension. Ethyl cellulose (EC) (Fig. 2a) was used as an organic binder, dispersing agent, and rheological modifier. In a typical ink formulation, EC was first mixed with terpineol and ethanol at a weight ratio of 1:4:0.4 to form a viscous solution. Then, the VO_2_ particles were mixed with the prepared solution at a weight ratio of 3:5, followed by agitation to obtain a homogenous and stable VO_2_ ink with a VO_2_ content of 37.5 wt.% (Fig. 2a). The viscosity of the resultant ink was measured using a rheometer at a shear rate from 0.1 to 1000 s^−1^, and the curve is displayed in Fig. 2b. We clearly observed a shear thinning behavior of the formulated VO_2_ ink. The viscosity decreased from 12,492 to 58 Pa s as the shear rate increased from 0.1 to 10 s^−1^. This type of fluid behavior and high viscosity are essential to provide favorable printability. This ink was directly used for screen printing to deposit VO_2_ switches on various substrates. What should be noted is that the single-pass thickness of the printed VO_2_ film was affected by many factors, such as the screen mesh parameters (i.e., mesh count, mesh diameter, and emulsion thickness), printing parameters (i.e., printing speed, squeegee pressure, and snap-off distance), and ink properties (i.e., solid content and viscosity)^47,48^. Typically, a smaller mesh count, thinner wire, thicker emulsion, slower speed, larger pressure, larger snap-off distance, more solid content, and higher viscosity print thicker films. However, among all these factors, the mesh count and the solid content (i.e., VO_2_ particles and EC) of the ink are critical for controlling the thickness of the printed films. Specifically, a smaller mesh count means fewer openings per inch and a larger size of each opening in the screen, which results in a thicker film. In our experiment, the mesh count was fixed as 325. Thus, the dominant factor controlling the single-pass thickness was the solid content. A low solid content results in a thin layer. However, this might produce additional pores in the film due to the high amount of solvent. As a result, the electrical performance (i.e., ON/OFF ratio) of the printed VO_2_ films worsened, as shown in Fig. S4. After considering the optimized ink formulation (i.e., solid content and viscosity) and the printing parameters (i.e., printing speed and mesh count), the resultant thickness of the printed VO_2_ film in a single pass was ~8–10 µm. After the screen-printing process, the printed VO_2_ switches were baked in an oven at 120 °C for 1 h to evaporate the solvents and sinter the VO_2_ film. It is noteworthy that the printed VO_2_ films without polymer binders contained particle aggregates and cracks after thermal sintering, as can be observed from the optical images in Fig. S5. The limited addition of the polymer binder (the weight ratio of VO_2_ to binder is ~3.3) was favorable for dispersing the VO_2_ particles and achieving flexibility of the printed VO_2_ film.

The low processing temperature enabled our VO_2_ ink to be suitable for various substrates, such as flexible polymers or even paper, largely widening its applications. Fig. 2c, d display digital photographs of the large-area VO_2_ switches printed on flexible Kapton and PET substrates in flat and bent states, respectively. We investigated the printed VO_2_ line features by comparing the printed line widths with the designed lines on the screen mesh. As presented in Fig. 2e, a linear behavior was observed between the printed and designed lines over the whole line width range (from 50 µm to 1.5 mm), demonstrating the excellent reliability of the screen-printed VO_2_ switches. The narrowest line width was ~65 µm. In addition, we found that the printed VO_2_ lines were slightly wider (~30 µm) than the designed lines, which was attributed to ink spreading after transfer to the substrates. We further printed a thick and large VO_2_ film on a 2-inch sapphire wafer with 15 printing passes to demonstrate high-throughput printing, as displayed in Fig. 2f. The printed VO_2_ film had a thickness of ~120 µm, as identified by the profile curve (inset in Fig. 2f), which was obtained by scanning the film with a profilometer (from the edge to the center). From the curve, we can also observe that the surface of the printed VO_2_ film was not smooth, which was attributed to the micron-sized particles. Notably, the printed wafer-scale VO_2_ film was of high quality, as confirmed by XRD pattern (Fig. S6), where four distinct diffraction peaks were indexed to pure VO_2_ (M) without any impurities. It should be noted that this film was printed at a high speed of 220 mm s^−1^, and it took only a few minutes to print the VO_2_ film. In comparison, tens of hours or even days are necessary to deposit the same VO_2_ film using the inkjet printing technique. All these results imply that our developed screen-printable VO_2_ ink and the conducted screen-printing process are ideal for mass production of VO_2_-based switches for tunable and reconfigurable RF electronics.

**Fig. 3:**
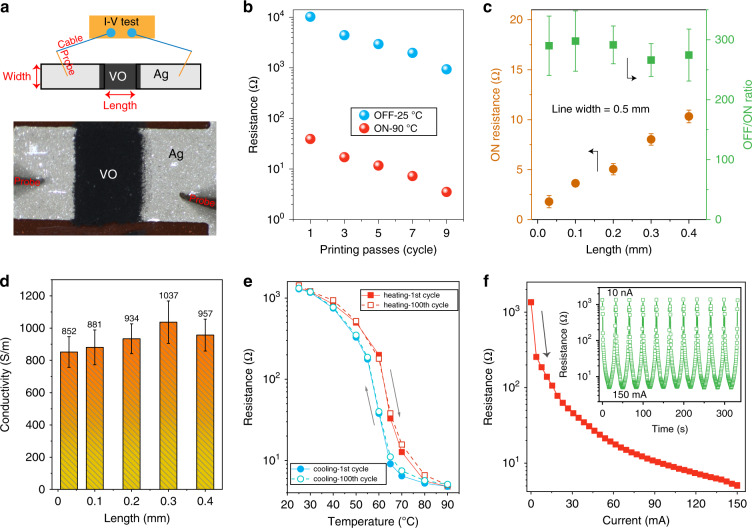
Electrical performance of the printed VO_2_ switches. **a** The setup diagram (top panel) to measure the resistance of the printed VO_2_ switch. Bottom panel: VO_2_ switch under test. **b** The measured resistance of the VO_2_ switches as a function of printing passes at 25 °C (OFF state) and 90 °C (ON state). **c** The measured ON resistance and ON/OFF ratio of the VO_2_ switches as a function of switch length. **d** The calculated electrical conductivity of the printed VO_2_ switches as a function of switch length. **e** The measured resistance of the printed VO_2_ switch at different temperatures in the heating and cooling process. **f** The measured resistance of the printed VO_2_ switch at different bias currents of 10 nA and 150 mA. Inset: the resistance as a function of cyclic electrical triggering

We then evaluated the electrical properties of the screen-printed VO_2_ switches. Figure 3a presents the apparatus for the current–voltage (I–V) measurements, in which two probe tips were connected to the samples and a source measurement unit to measure the resistance of the VO_2_ switches. VO_2_ was printed between two silver (Ag) electrodes. The electrode width and the gap between the electrodes were marked as the width and length of the VO_2_ switches, respectively. Figure 3b displays the measured resistance of the printed VO_2_ switches (width and length were 0.5 and 0.3 mm, respectively) with increasing printing passes from one to nine at 25 °C (called the OFF state) and 90 °C (called the ON state). The resistance at both the OFF and ON states decreased rapidly for a larger printing pass, as a thicker and denser VO_2_ layer was formed. Specifically, the OFF resistance was 1976 ohm, while the ON resistance was 7.3 ohms after seven printing passes, yielding an ON/OFF ratio of 270, which is much higher than that of inkjet-printed VO_2_ films^12,17^. The reason for this was attributed to the (1) high quality of the VO_2_ particles, as confirmed by the intense XRD peaks; (2) well-dispersed VO_2_ particles in the ink system; and (3) dense VO_2_ layer via polymer binding. We then investigated the resistance of the VO_2_ switches with various lengths. As presented in Fig. 3c, when the VO_2_ film width was fixed as 0.5 mm, the measured ON resistance linearly increased from 1.8 to 10.3 ohms as the VO_2_ length increased from 0.035 to 0.4 mm, respectively. Thus, VO_2_ switches with varying ON and OFF resistances (within the bound of the best-case scenarios) can be designed by selecting different geometric parameters of the VO_2_ film, such as length, width, and thickness. The electrical conductivity (*σ*) of the printed VO_2_ switches in the ON state was calculated based on the measured ON resistance (*R*) and the VO_2_ geometries, such as VO_2_ length (*l*), VO_2_ width (*w*), and VO_2_ thickness (*t*), through the following equation, *σ* = *l*/(*Rwt*). The measured thicknesses and the calculated conductivities are displayed in Fig. S7 and Fig. 3d, respectively. Various samples for each length were measured, and an average conductivity value was obtained for each length. Generally, the average conductivity values in the ON state for all the samples are almost similar with some minor fluctuations (in the range of 852 to 1037 S m^−1^). These values are superior to the value of 200 S m^−1^ obtained for inkjet-printed VO_2_ films^12^. The slight fluctuation in the calculated conductivity can be attributed to fabrication tolerances because the samples with different lengths had slightly different thicknesses and widths. Furthermore, the edges of printed films are typically not smooth, and the surfaces are relatively rough, which can further affect the calculated results. Nevertheless, such a high ON/OFF ratio and electrical conductivity are promising for printed VO_2_ films that have been reported. Note that the achieved ON/OFF ratio (~10^2^) is low compared to the typical values achieved from the VO_2_ films deposited through nanofabrication techniques (~10^4^) due to the irregular VO_2_ particles during synthesis, partially covering of VO_2_ particles by polymer binders (Fig. S8), and the pores in the printed films. However, printed films have the advantages of easy processing, direct patterning, and roll-to-roll production capability. With the advancement of printing techniques and inks, it is expected that in the near future, printed films will have a comparable performance to films deposited through nanofabrication techniques.

As a material with an MIT, VO_2_ could be stimulated by various external stimuli. Here, we evaluated the electrical response of the printed VO_2_ switches by applying heat and a bias current. We selected a typical VO_2_ switch for further study that had a length of 0.2 mm, a width of 0.5 mm, and an ON resistance of ~5 ohms. As displayed in Fig. 3e, at room temperature (25 °C), the measured resistance was ~1300 ohms, exhibiting insulating behavior. Then, the resistance slowly decreased to 200 ohms as the temperature increased to 60 °C. When the temperature reached 65 °C, the resistance dropped dramatically to 33 ohms, exhibiting an 83.5% decrease compared with the resistance at 60 °C. This is consistent with the transition temperature of 65.6 °C observed in the DSC result (Fig. 1e). With a further increase in the temperature, the resistance decreased to 5.5 ohms at 80 °C and remained constant at a value of approximately 5 ohms at 90 °C. During the cooling process, the resistance increased from 5 ohms to ~1300 ohms when the temperature decreased from 90 °C to 25 °C, respectively. Notably, this resistance change with temperature was highly repeatable, as the hundredth heating and cooling of the same sample demonstrated a similar electrical response in both the trend for the resistance change and the OFF and ON resistances, despite a slight variation in some of the resistance values. A similar resistance response was observed by applying different bias currents to the VO_2_ switches, as presented in Fig. 3f. The initial resistance at an applied current of 10 nA was ~1351 ohms, which was in the OFF state. The resistance dropped dramatically to 254 ohms at a supplying current of 3.6 mA and finally reduced to 5.1 ohms when the current was 150 mA. As the current further increased to 160 mA, the VO_2_ switch was burned, implying that careful selection of the applied current is important and that the current should be increased step by step. Similar to heat triggering, the resistance response of the VO_2_ switches with electrical triggering demonstrated excellent repeatability via the cyclic application of currents of 10 nA and 150 mA (inset in Fig. 3f). What should be noted is that the geometric parameters of a VO_2_ switch, such as its length, width, and thickness, determine the biasing current applied to the switch. Typically, a wider, longer, and thicker VO_2_ switch requires a higher biasing current^12,29^.

**Fig. 4:**
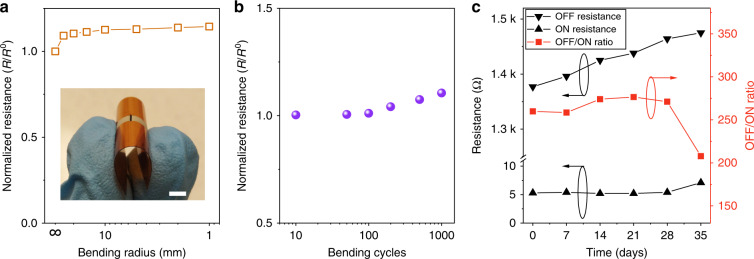
The mechanical and air stability of the printed VO_2_ switches. **a** The normalized resistance of the VO_2_ switch under different bending radii. Inset: digital photograph of the VO_2_ switch under bending. The scale bar is 5 mm. **b** The normalized resistance of the VO_2_ switch under cyclic bending with bending radii from infinite to 1 mm. **c** The measured resistance and ON/OFF ratio of the VO_2_ switch at different times after environmental exposure

The printed VO_2_ switches not only demonstrated a high ON/OFF ratio, electrical conductivity, and triggering repeatability but also presented excellent flexibility and mechanical stability. We evaluated the resistance change of a typical printed VO_2_ switch by applying various bending radii and repeated bending cycles. As presented in Fig. 4a, the resistance of the VO_2_ film with a bending radius of 25 mm increased by 10% compared with that in its flat state (infinite radii), which was attributed to tensile release. As the bending radii decreased, the resistance became relatively stable and exhibited negligible degradation (~4%), even when the radius was reduced to 1 mm. In addition, the resistance was retained even under repeated bending of 1000 cycles with a bending radius from infinite to 1 mm, though an 11% increase in resistance was observed after the bending test, as depicted in Fig. 4b. This excellent mechanical stability was attributed to the polymer binders between the VO_2_ particles, which largely enhanced the particle interconnections and provided a fixed network to diminish film cracks, enabling a reliable VO_2_ switch for practical use. In addition to the flexibility, we evaluated the long-term stability of the printed VO_2_ switches by examining the resistance changes in a typical indoor environment (temperature: 25 °C, humidity: 55%). As depicted in Fig. 4c, the VO_2_ switches exhibited outstanding durability, in which the ON resistance was constant even after exposure to air without any protection for one month. The slightly increased ON/OFF ratio was due to an increase in the OFF resistance, which was attributed to the absorbed moisture on the surface of the VO_2_ switches. The OFF resistance recovered to its initial value after heating at 100 °C for 10 min. After one month, the ON resistance began to increase, which caused a sharp decrease in the ON/OFF ratio. Nevertheless, our printed VO_2_ switches demonstrated highly stable behavior under mechanical deformation and in air, which is essential and necessary for practical applications.

**Fig. 5:**
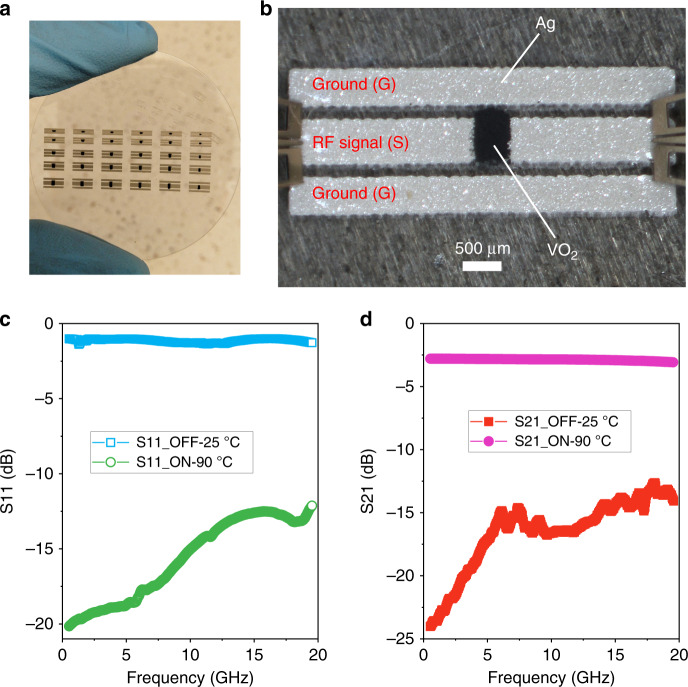
The RF performance of the printed VO_2_ series switches. **a** Digital photograph of the fully screen-printed series switches on a sapphire wafer. **b** Optical image of one series switch under the RF performance test. **c** The measured S11 of the series switch at 24 °C and 90 °C. **d** The measured S21 of the series switch at 24 °C and 90 °C

In addition to the electrical properties of the printed VO_2_ switches, the RF performance is another critical factor that was investigated for potential applications in reconfigurable RF electronics; specifically, a high RF isolation and transmission in the OFF and ON states, respectively, are important. We first printed a coplanar waveguide (CPW)-based series switch on a sapphire wafer using Ag ink (Fig. 5a). The series switch contained two ground lines separated by one signal line; a gap was left in the middle of the signal line to print the VO_2_ switch (width: 500 µm, length: 200 µm) (Fig. 5b) that acted as a switch to control the RF signal being transmitted between the two ends of the signal line. In the OFF state, the VO_2_ switches were deactivated, and thus, the resistance of the VO_2_ switch was high enough (~1500 ohm) to block the RF signal for transmission. In contrast, the RF signal could transmit in the signal line when the resistance of the VO_2_ switch was low and in the activated status (~5 ohm). The RF performance for the printed VO_2_ switches was examined by investigating the scattering parameters (S-parameters S11, S12, S21, and S21) of the series switch via a two-port measurement (Fig. 5b). Parameters S11 and S22 refer to the RF signal reflection at port 1 and port 2, respectively, which were identical in the measurement. To achieve perfect isolation, the RF signal was totally reflected, and the measured S11 (or S22) was 0 dB. Similarly, S12 and S21 indicated the RF signal transmission from port 1 to port 2 and from port 2 to port 1, respectively, which were coincident with each other. For an ideal transmission, the RF signal is completely transmitted from one port to the other, and the measured S21 (or S12) should be 0 dB. For our screen-printed series switches, at room temperature (OFF state), the measured S11 was approximately −1 dB over the entire measured frequency range up to 20 GHz (Fig. 5c), indicating satisfactory isolation. This was also confirmed by the S21 measurement, in which less than −13 dB was observed. In contrast, S11 became lower than −12 dB, and S21 increased to −2.6 dB (also called an insertion loss) when the temperature was 90 °C, demonstrating a reasonable transmission of the RF signal. These results indicated that our screen-printed VO_2_ switches demonstrated decent RF performance with an acceptable value of −2.6 dB for an RF switch, though it is larger than a typical value of −1.5–2.0 dB for a nonprinted VO_2_ switch through nanofabrication techniques^29^. To further identify this, a band-stop filter was fabricated on glass, and the RF performance was characterized. As illustrated in Fig. S9a in the Supporting Information, the filter was a microstrip-based T resonator and contained three VO_2_ switches to adjust the length and width of the stub, enabling the tunability of both the resonant frequency and bandwidth. Here, two frequencies, 1.9 GHz (GSM) and 2.4 GHz (Bluetooth) were selected as a proof of concept, while any other frequency could be selected by simply changing the electrical length of the open stub in the T resonator. One prototype is presented in Fig. S9b that contained a fully printed filter and a printed heater element to activate the VO_2_ switches. The RF performance was measured using a vector network analyzer E8363C from Agilent. Fig. S9c–e presents the measured S-parameters (S11 and S21) of the filter under three different operating conditions in accordance with the three working conditions of VO_2_ switches. Table S1 in the Supporting Information summarizes the detailed measurement results. We observed well-matched results between the measurement and simulation. The resonant frequency of the filter shifted from 2.4 GHz to 1.9 GHz when switch one was ON. The bandwidth was considerably widened from 320 to 500 MHz at 2.4 GHz when switch two was ON and from 200 to 520 MHz at 1.9 GHz when all three switches were in the ON state. These results matched well with the simulation, providing confirmation of the reliable RF performance of our screen-printed VO_2_ switches.

**Fig. 6:**
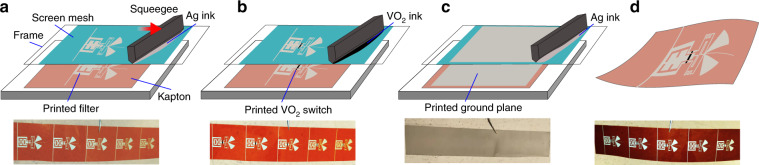
Schematic illustration of fully screen-printed reconfigurable band stop filters. **a** Screen printing of the filters with Ag ink. **b** Screen printing of the VO_2_ switches with VO_2_ ink. **c** Screen printing of the ground plane with Ag ink. **d** The fabricated filter

The excellent electrical performance, together with decent RF performance, makes our screen-printable VO_2_ ink suitable for high-performance, tunable, and reconfigurable RF electronics. As a proof of concept, we demonstrated the high-throughput production of fully screen-printed and flexible band-pass filters on a Kapton film. Figure 6 illustrates the fabrication steps, which consist of (a) screen-printing of band-pass filters on the Kapton film; (b) screen-printing of VO_2_ switches in the desired areas; and (c) screen-printing of the ground plane. The printed samples in each step are also shown in the bottom panel of Fig. 6, in which six filters were produced in one cycle to improve the fabrication efficiency. For convenience, a common ground plane was printed for all the switches, as shown in Fig. 6c. However, for the measurements, each filter was cut separately to avoid any electrical connection. A photograph of the fabricated filters is displayed in Fig. 6d.

**Fig. 7:**
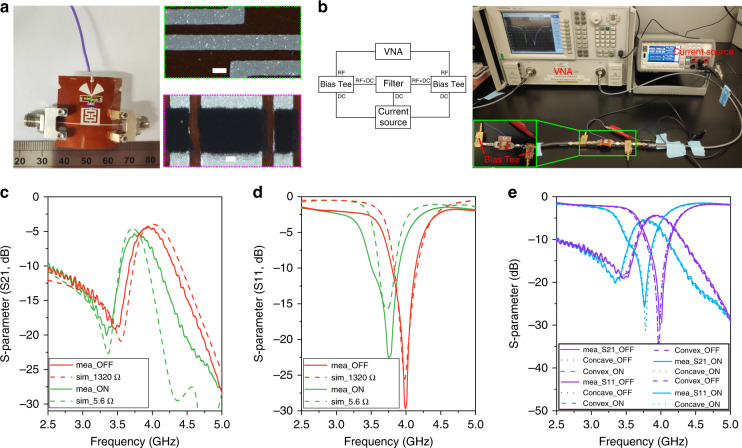
Performance of the fully screen-printed, flexible band-pass filters. **a** Digital photograph of one filter (left panel) with stimulating DC line and RF connectors. Right panel: optical images of the printed Ag lines and VO_2_ switches. Scale bars are 200 µm. **b** Schematic illustration (left panel) and digital photograph of the measurement apparatus. **c** The measured and simulated S21 of the filter at the OFF and ON states. **d** The measured and simulated S11 of the filter at the OFF and ON states. **e** The measured S11 and S21 of the filter at the OFF and ON states under various bending conditions

As presented in Fig. S10 in the Supporting Information, the designed filter contains three parts: a main body (marked as dark gray), VO_2_ switches (marked as red), and a triggering component (marked as light gray). The three switches were used to tune the length of the filter, allowing an RF signal pass through at a center frequency of 4 GHz with a 500 MHz bandwidth at the OFF state, while the working frequency shifted to 3.75 GHz when the switch was ON. The triggering lines were designed to inject bias current to activate the VO_2_ switches. The detailed geometrical dimensions of the designed filter and the 3D model in the full-wave simulator are depicted in Fig. S10. Furthermore, Fig. 7a presents one filter prototype ready to test, which was connected with two subminiature version A connectors to transmit an RF signal and a DC wire (color in purple) to apply current. The optical images of the printed Ag lines and VO_2_ switches are displayed in the right panel of Fig. 7a, demonstrating fairly clear and smooth edges that enable the stability and reproducibility of RF electronic devices. A diagram and digital photograph of the apparatus to measure the S-parameters of the fabricated filter is presented in Fig. 7b. Each end of the filter was connected to a bias Tee, which combined the RF signal from a precalibrated Vector Network Analyzer (VNA) with the DC current from a current source to provide an RF signal and bias current to the filter. The S-parameters were recorded by the VNA and replotted in Fig. 7c, d, together with the simulation. If no current was applied, the three VO_2_ switches were in the OFF state. In this case, the triggering component was disconnected from the main body of the filter, resulting in a shortened filter resonating at a high frequency, i.e. 4 GHz. The measured S21 exhibited a center frequency of 3.95 GHz with an insertion loss of 4.3 dB, while an operating frequency of 4 GHz with an insertion loss of 3.9 dB was obtained from the simulation. The pass-band widths (frequencies at 3 dB attenuation) were 0.45 and 0.48 GHz for the measurement and simulation, respectively. Similar results were also observed from the S11 curves, as presented in Fig. 7d. In contrast, the three VO_2_ switches were activated in the ON state when the DC current (200 mA) was injected into the filter through the triggering component. Correspondingly, the triggering component was connected to the filter body, which resulted in a long filter operating at a low frequency (i.e., 3.75 GHz). As presented in the S21 curves in Fig. 7c, the center frequency for the measurement and simulation were 3.77 and 3.72 GHz, respectively, while there was a 0.7 dB difference in the insertion loss. Furthermore, we observed that the pass-band widths were 0.42 GHz and 0.31 GHz for measurement and simulation, respectively. The resonant frequencies in the S11 curves demonstrated similar results. Although the measured RF performance of the fabricated filter matched well with the simulated results, minor shifts in the center frequency, mismatched bandwidth, and rippled curves were observed. This could be attributed to several reasons: (1) the printed Ag lines were slightly wider than the designed ones due to the ink spreading on the substrate, as confirmed by Fig. 2e; (2) the surfaces of the printed Ag lines and VO_2_ layers were not smooth (Fig. S11a, b), resulting in possible wave diffraction; (3) the polymer binders in the Ag and VO_2_ layers (Figs. S11c and S7) negatively affected the RF performance; and (4) the calibration tolerance. We then concluded by investigating the flexibility of the fabricated filter by measuring the S-parameters under bending conditions, i.e., convex and concave directions. Figure 7e displays the measured results of S11 and S21, in which we found well-matched curves for various bending conditions in both the OFF and ON states, demonstrating consistent performance during mechanical deformation and supporting potential applications in flexible RF electronic devices.

## Discussion

In summary, a screen-printable VO_2_ ink was developed by dispersing high-purity VO_2_ microparticles into a viscous EC matrix. VO_2_ switches with uniform and clear edges were printed with the ink on flexible polymers and rigid wafers over a large area and with a high efficiency (up to 220 mm s^−1^). A low annealing temperature of 120 °C was used to sinter the printed VO_2_ switches to achieve a low ON resistance (as low as 1.8 ohm), high electrical conductivity (larger than 1000 S m^−1^), and excellent ON/OFF ratio (more than 300). Both thermal and electrical triggers were successfully used as stimuli to trigger the VO_2_ switches. When integrated into CPW microstripe lines, the screen-printed VO_2_ switch controlled the transmission of the RF signal, demonstrating a −1 dB isolation and −2.6 dB transmission at 25 °C (OFF state) and 90 °C (ON state), respectively. The VO_2_ switches were successfully integrated into fully screen-printed frequency reconfigurable RF electronics (i.e., band-stop filter and band-pass filter). A batch of six band-pass filters was printed on Kapton film in one printing process. The center frequency of the filters was tuned from 3.95 to 3.77 GHz after applying a DC current of 200 mA via the triggering component. Moreover, the measured S-parameters were consistent under flat and bending conditions, implying high mechanical stability. All these results present a promising approach for realizing reconfigurable RF electronics through a fully screen-printed process.

## Materials and methods

### Chemicals

Vanadium (V) oxide (V_2_O_5_, ≥ 98%, Sigma-Aldrich), oxalic acid dihydrate (HO_2_CCO_2_H·2H_2_O, ≥ 99%, ACS reagent, Sigma-Aldrich), ethyl cellulose (EC, Sigma-Aldrich), terpineol (ACS reagent, Sigma-Aldrich), and ethanol (>99%, Sigma-Aldrich) were used as received without further purification.

### Synthesis of VO_2_ microparticles

In a typical synthesis process, 3 g oxalic acid was dissolved in 150 mL deionized (DI) water, and 0.5 g of vanadium (V) oxide powder was subsequently added. The resultant mixture was mixed well until becoming a yellow-colored slurry solution. The final solution was then transferred into a 200 mL PPL high‐temperature polymer‐liner‐based hydrothermal autoclave reactor. The reaction temperature was set to 240 °C. Three sets of experiments were performed, varying the reaction times from 3–24 h. After the completion of the reaction, the resultant blue-black precipitate was centrifuged, washed with water and ethanol and then dried in a vacuum furnace at 70 °C for 1 h. The obtained dried powder was then annealed at 300 °C for 3 h in a vacuum furnace to obtain VO_2_ (M) microparticles.

### Ink formulation and screen printing

First, a viscous organic binder solution was formulated by mixing EC, terpineol, and ethanol at a weight ratio of 1:4:0.4. Prior to printing, the obtained pure VO_2_ particles were mixed with the prepared solution at a weight ratio of 3:5, followed by agitation to obtain the VO_2_ ink. Screen printing was performed on a professional printing system (AUREL screen printer 900PA) with a 21 inch × 21 inch stainless-steel screen mesh mask (325 mesh count, 22.5° mesh angle, 10 µm emulsion thickness) at a printing speed of 220 mm s^−1^. The VO_2_ switches were printed at the gaps between the silver traces, which were screen printed using commercial silver paste (PE819, DuPont) and annealed at 300 °C for 1 h to obtain a conductivity of 1.8 × 10^7^ S m^−1^. Finally, the printed VO_2_ switches were sintered at 120 °C for 1 h.

### Filter simulation

A professional simulator (CST Microwave Studio 2019) was used for the full-wave simulation, and the constructed 3D model of the filter is presented in Fig. S10. The silver traces were modeled as a sheet with a conductivity of 1.8 × 10^7^ S m^−1^, a thickness of 8 µm, and a surface roughness of 1 µm. The dielectric substrate had a permittivity of 2.8 ± 0.02j and a thickness of 125 µm. The VO_2_ switches were modeled as lumped elements with resistances of 5.6 ohms and 1320 ohms. The two sources were molded as a discrete 50-ohm port. The frequency range was set at 2.5–5 GHz.

### Characterization

The morphologies of the VO_2_ particles were characterized by scanning electron microscopy (ZEISS Merlin) and transmission electron microscopy (FEI Titan G2). The structure of the VO_2_ particles was characterized by XRD (Bruker D2 PHASER). The DSC was performed using the Discovery DSC tool. The rheological behavior of the formulated VO_2_ ink was tested using an AR1500 rheometer (TA Instruments). The thickness of the VO_2_ film was measured using a surface profiler (Veeco Dektak 150). The RF measurements were performed using an Agilent N5225A Vector Network Analyzer. The S-parameters of the filters were acquired from 2.5 to 5 GHz after RF calibration using the standard short-open-load (SOL) method to eliminate parasitic effects of the measurement system.

## Supplementary information

Supplementary Information

## Data Availability

The datasets generated during and/or analyzed during the current study are available from the corresponding author on reasonable request.
