# Supplementary material for: Flexible and reconfigurable radio frequency electronics realized by high-throughput screen printing of vanadium dioxide switches
Source: Microsyst Nanoeng. 2020 Oct 5;6:77. doi: 10.1038/s41378-020-00194-2 (PMC8433205; doi:10.1038/s41378-020-00194-2)
Supplement: Supplementary file 1 — Supplementary Information [file 41378_2020_194_MOESM1_ESM.docx]

Supplementary Information

Flexible and Reconfigurable Radio Frequency Electronics Realized by High-Throughput Screen Printing of Vanadium Dioxide Switches

Weiwei Li^🕆,1^, Mohammad Vaseem^🕆,1^, Shuai Yang^1^, Atif Shamim^1^

^1^IMPACT Lab, Computer, Electrical and Mathematical Sciences and Engineering (CEMSE) Division, King Abdullah University of Science and Technology (KAUST), Thuwal 23955-6900, Kingdom of Saudi Arabia


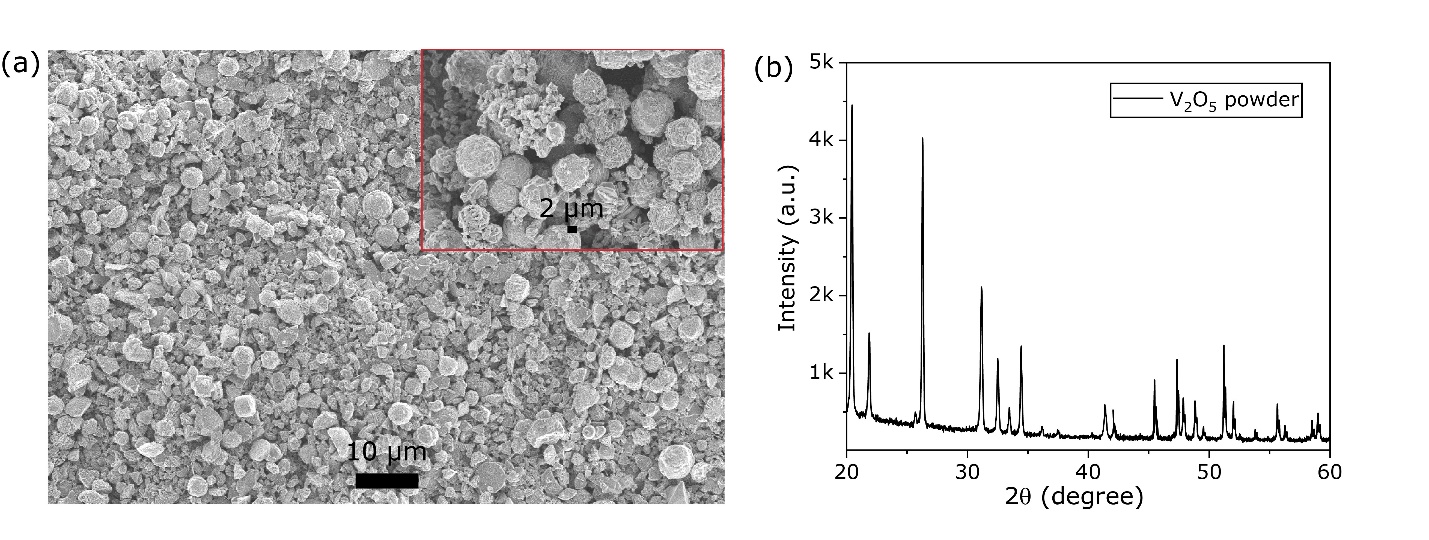


Figure S1. (a) SEM images and (b) XRD spectra of the V_2_O_5_ particles.


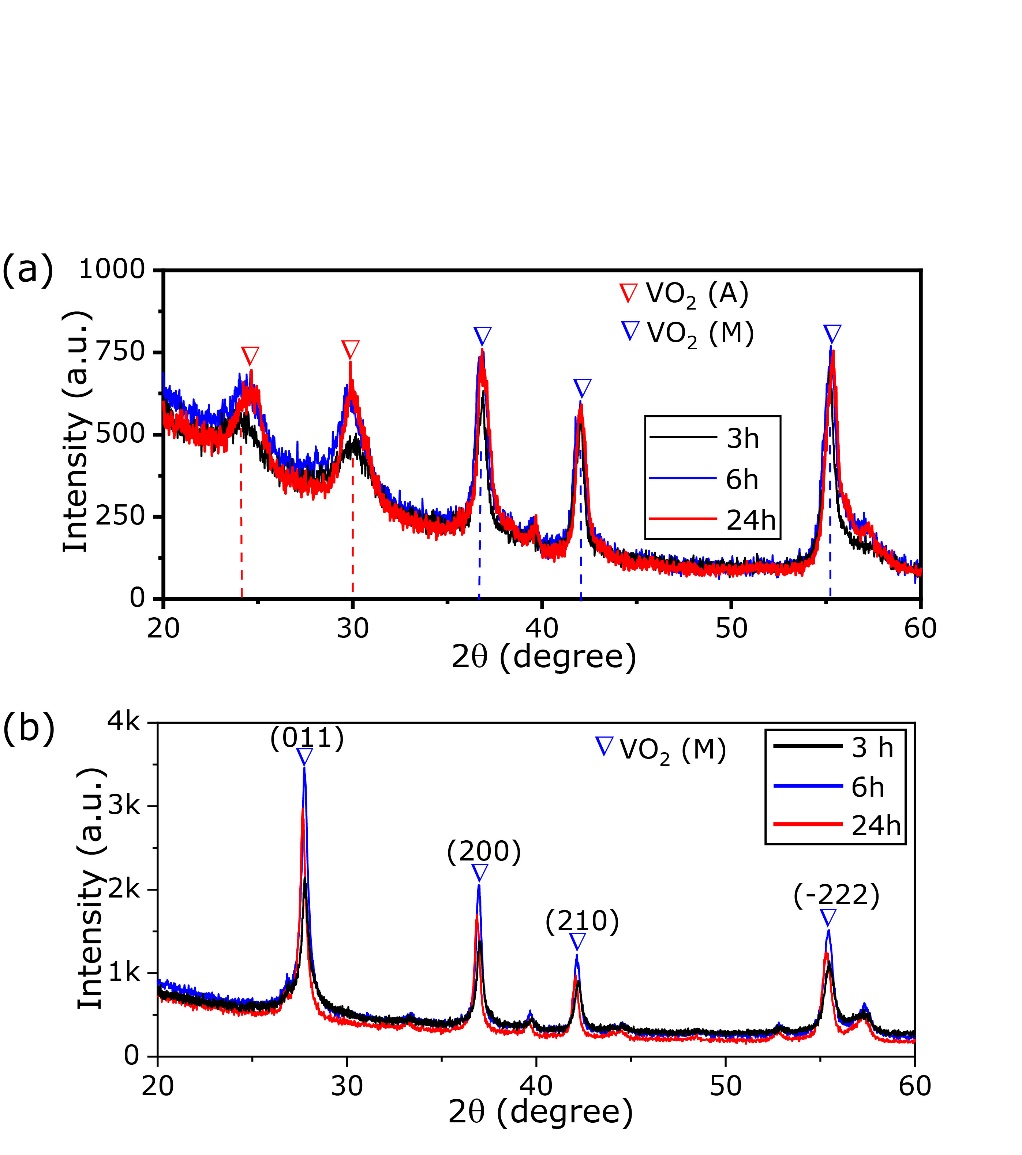


Figure S2. XRD spectra of the as-synthesized VO_2_ particles with different reaction times before annealing at 300 °C in vacuum.


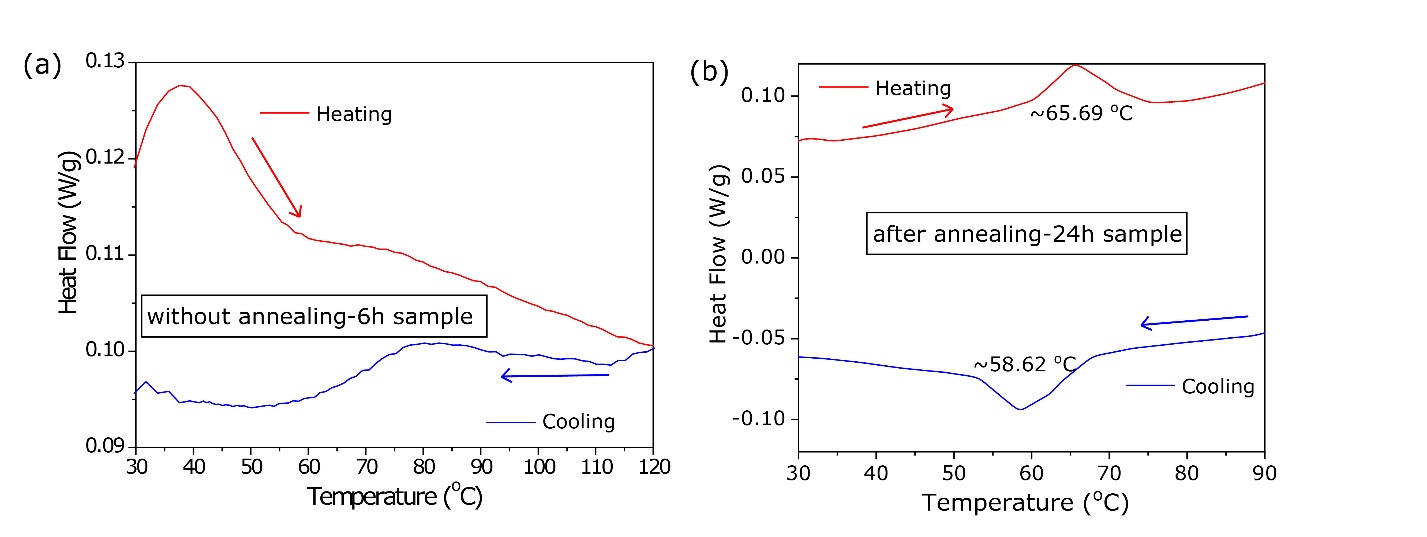


Figure S3. DSC spectra of the synthesized VO_2_ particles before (a) and after (b) annealing at 300 °C in vacuum.


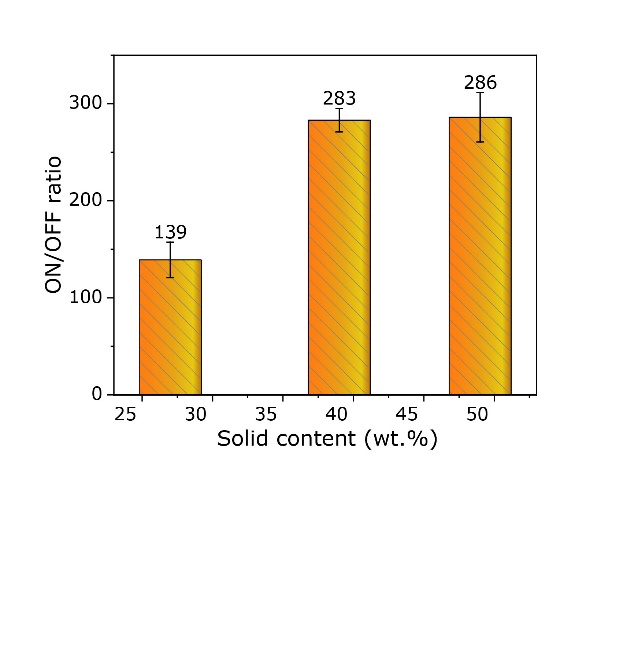


Figure S4. The measured ON/OFF ratio of the printed VO2 films with different solid contents.


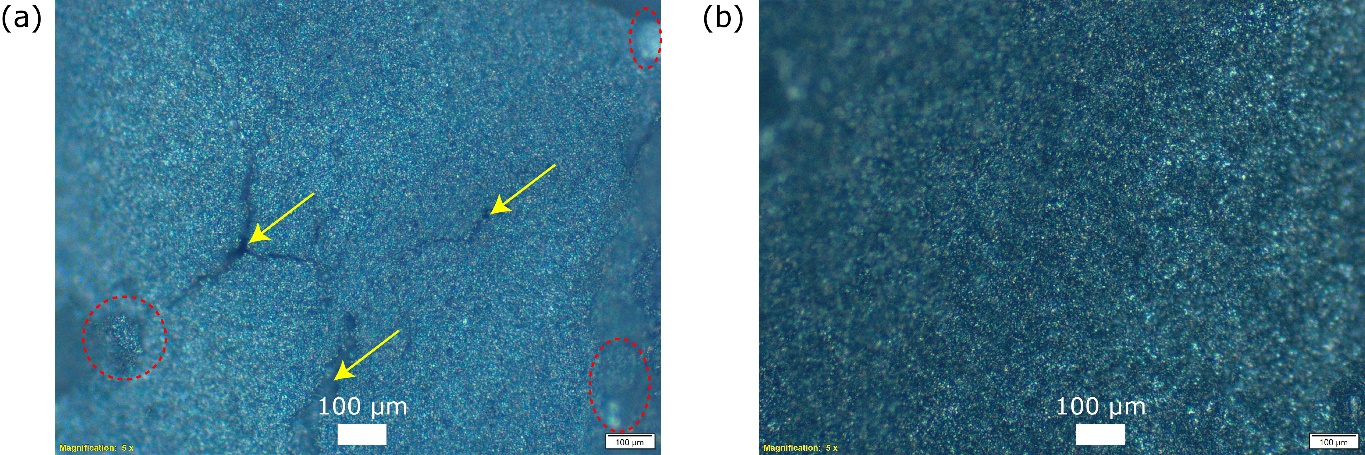


Figure S5. Optical images of the printed VO_2_ films without (a) and with (b) binder. The yellow arrows and red circles indicate the cracks and aggregated particles, respectively.


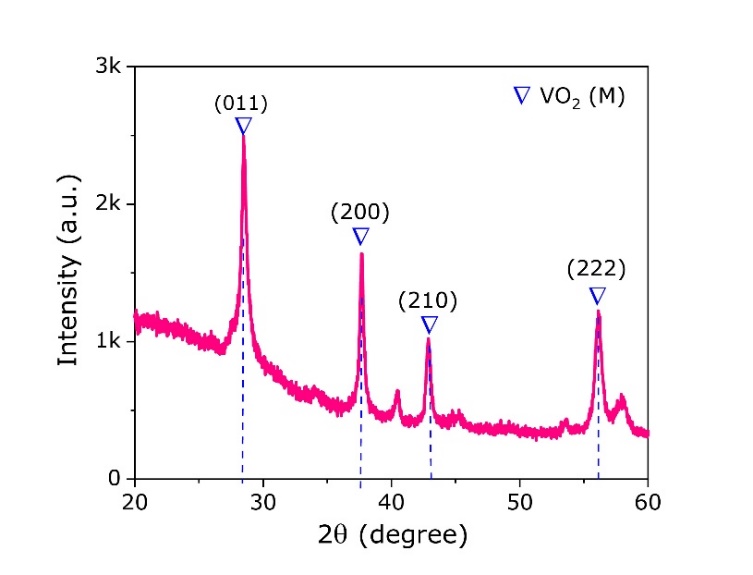


Figure S6. XRD pattern of the printed VO_2_ film on a 2-inch sapphire wafer.


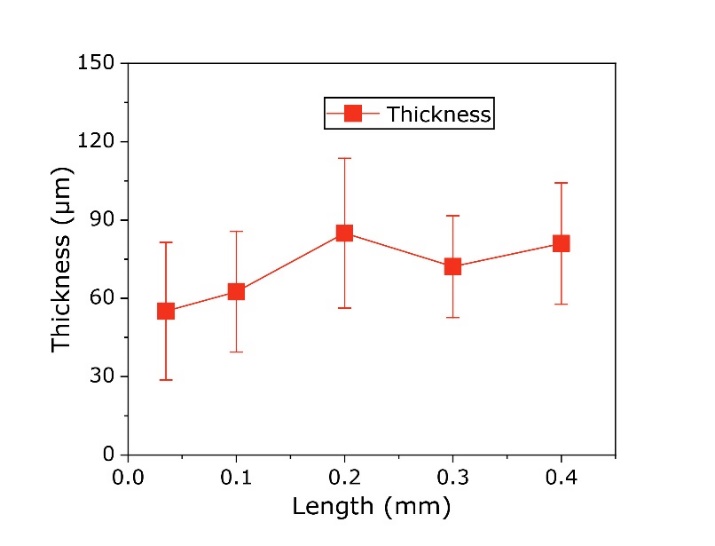


Figure S7. The measured thickness of the printed VO_2_ film with different VO_2_ lengths.


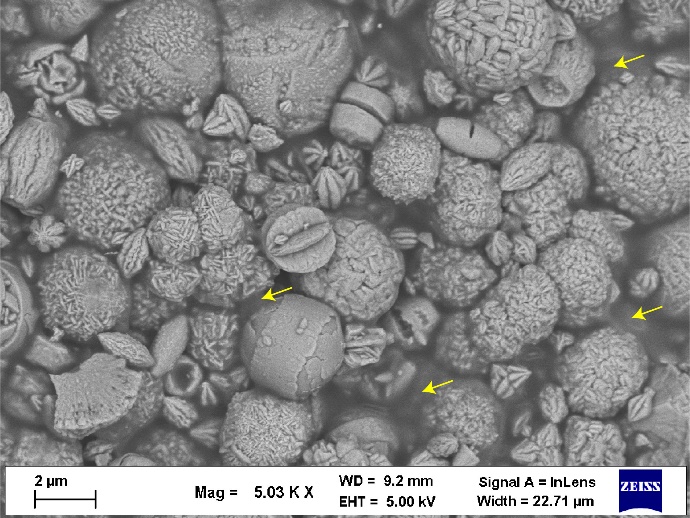


Figure S8. SEM image of the printed VO_2_ film. The yellow arrows indicate the polymer binders between the particles.


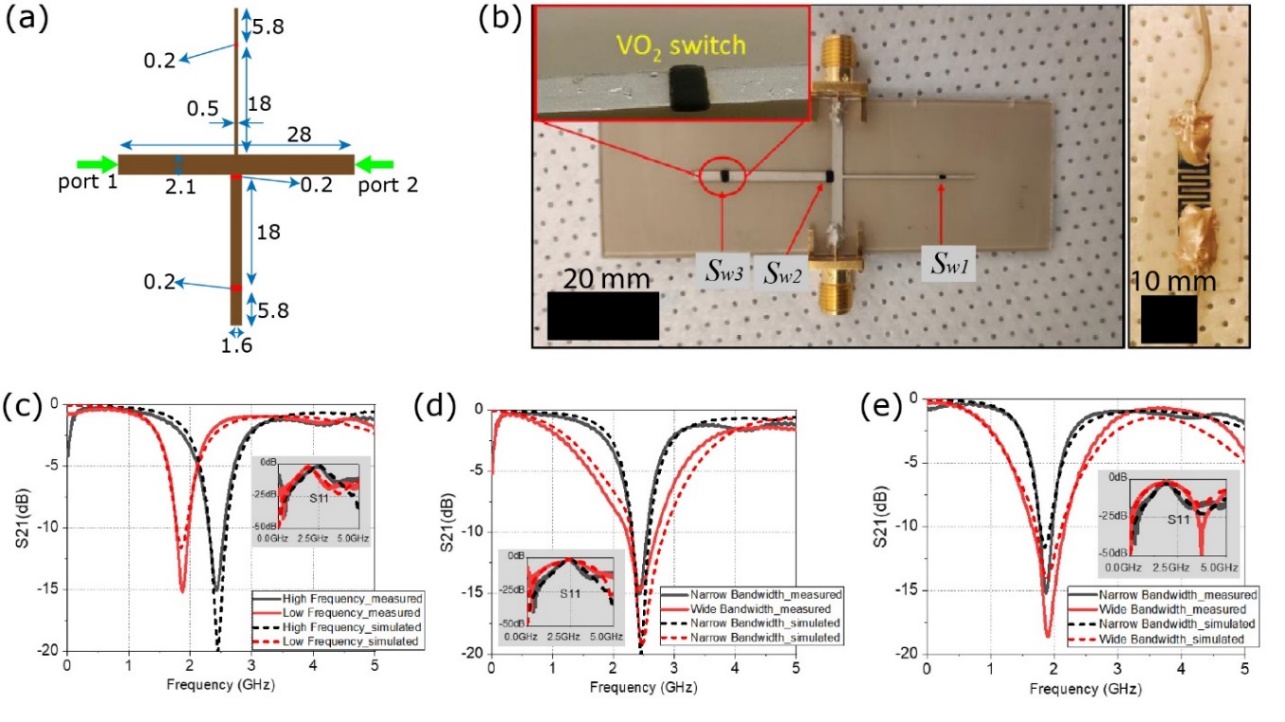


**Figure S9**. (a) The layout of the designed frequency and bandwidth-reconfigurable band-stop filter. All the dimensions are in millimeter. (b) The fabricated reconfigurable band-stop filter prototype and the printed heater. Three switches are marked as *S_w1_*, *S_w2_* and *S_w3_*. The simulated and measured S21 response of the band-stop filter with (c) *S_w1_* at the ON state, (d) *S_w2_* at the ON state, and (e) all three switches at the ON state. Insets: the S11 response for each combination.


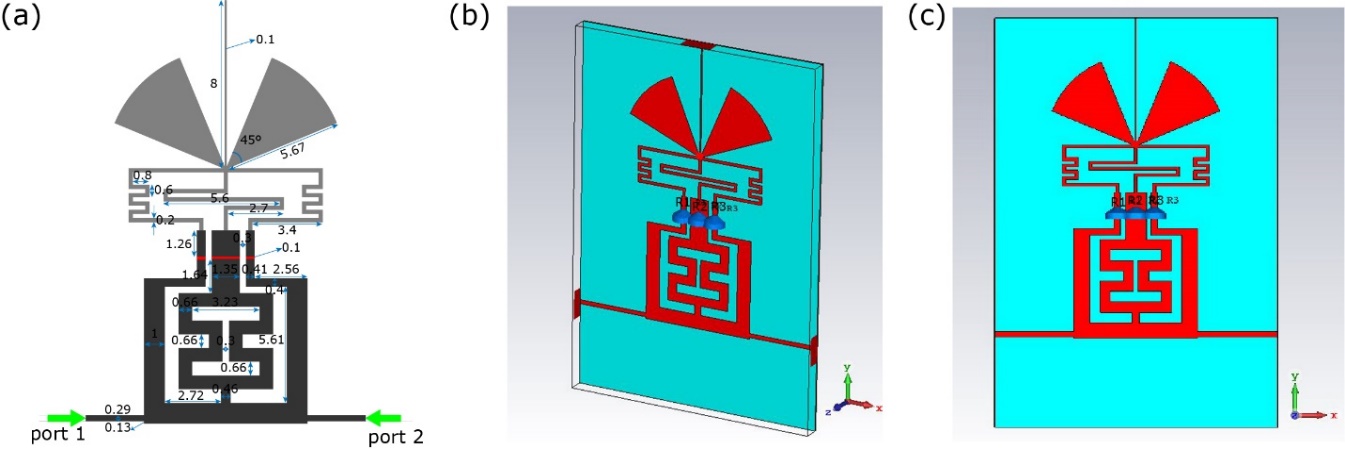
**Figure S10**. (a) Layout of the designed band-pass filter. All dimensions are in millimeters. (b) Perspective view and (c) front view of the 3D model of the designed filter in the CST simulator. The red and cyan parts are metallic traces and substrates, respectively. R1, R2, and R3 are VO_2_ switches.


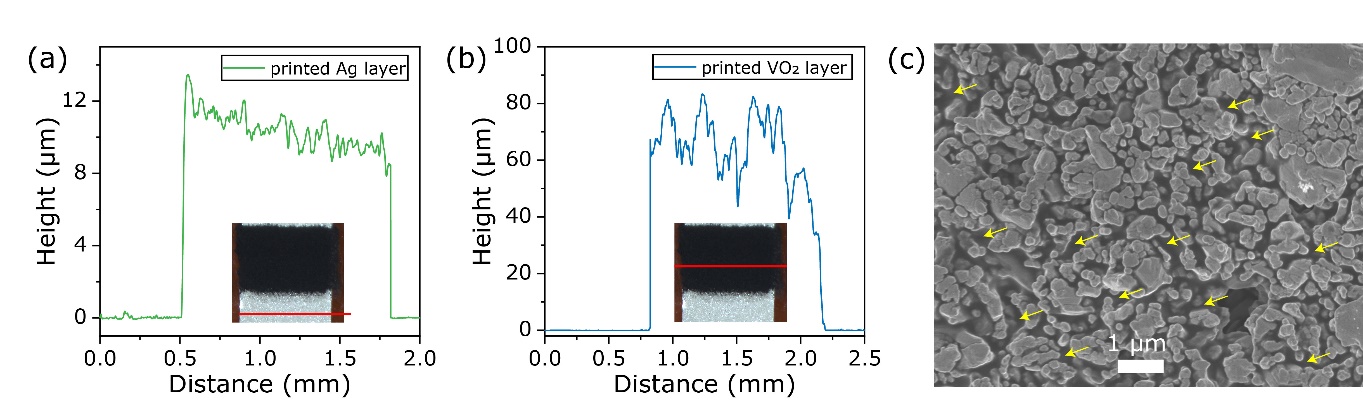


**Figure S11**. The measured height of the (a) printed Ag trace and (b) VO_2_ switch. Insets: optical images of the Ag trace and VO_2_ switch. The red lines are the scanning targets. (c) SEM image of the printed Ag trace. The yellow arrows indicate the polymer binders among particles.

**Table S1**. Summary of the measured results with different switch combinations of the band-stop filter.

|  | Switch combination | | | |
| --- | --- | --- | --- | --- |
|  | All OFF | *S_w1_* ON | *S_w2_* ON | All ON |
| Center frequency (GHz) | 2.4 | 1.9 | 2.4 | 1.9 |
| Bandwidth defined on -10 dB (GHz) | 0.3 | 0.2 | 0.5 | 0.5 |
